# Use of Radiomics Combined With Machine Learning Method in the Recurrence Patterns After Intensity-Modulated Radiotherapy for Nasopharyngeal Carcinoma: A Preliminary Study

**DOI:** 10.3389/fonc.2018.00648

**Published:** 2018-12-21

**Authors:** Shuangshuang Li, Kongcheng Wang, Zhen Hou, Ju Yang, Wei Ren, Shanbao Gao, Fanyan Meng, Puyuan Wu, Baorui Liu, Juan Liu, Jing Yan

**Affiliations:** The Comprehensive Cancer Centre of Drum Tower Hospital, Medical School of Nanjing University and Clinical Cancer Institute of Nanjing University, Nanjing, China

**Keywords:** intensity-modulated radiotherapy, nasopharyngeal carcinoma, recurrence pattern, radiomic analysis, prediction

## Abstract

**Objective:** To analyze the recurrence patterns and reasons in patients with nasopharyngeal carcinoma (NPC) treated with intensity-modulated radiotherapy (IMRT) and to investigate the feasibility of radiomics for analysis of radioresistance.

**Methods:** We analyzed 306 NPC patients treated with IMRT from Jul-2009 to Aug-2016, 20 of whom developed with recurrence. For the NPCs with recurrence, CT, MR, or PET/CT images of recurrent disease were registered with the primary planning CT for dosimetry analysis. The recurrences were defined as in-field, marginal or out-of-field, according to dose-volume histogram (DVH) of the recurrence volume. To explore the predictive power of radiomics for NPCs with in-field recurrences (NPC-IFR), 16 NPCs with non-progression disease (NPC-NPD) were used for comparison. For these NPC-IFRs and NPC-NPDs, 1117 radiomic features were quantified from the tumor region using pre-treatment spectral attenuated inversion-recovery T2-weighted (SPAIR T2W) magnetic resonance imaging (MRI). Intraclass correlation coefficients (ICC) and Pearson correlation coefficient (PCC) was calculated to identify influential feature subset. Kruskal-Wallis test and receiver operating characteristic (ROC) analysis were employed to assess the capability of each feature on NPC-IFR prediction. Principal component analysis (PCA) was performed for feature reduction. Artificial neural network (ANN), k-nearest neighbor (KNN), and support vector machine (SVM) models were trained and validated by using stratified 10-fold cross validation.

**Results:** The median follow up was 26.5 (range 8–65) months. 9/20 (45%) occurred in the primary tumor, 8/20 (40%) occurred in regional lymph nodes, and 3/20 (15%) patients developed a primary and regional failure. Dosimetric and target volume analysis of the recurrence indicated that there were 18 in-field, and 1 marginal as well as 1 out-of-field recurrence. With pre-therapeutic SPAIR T2W MRI images available, 11 NPC-IFRs (11 of 18 NPC-IFRs who had available pre-therapeutic MRI) and 16 NPC-NPDs were subsequently employed for radiomic analysis. Results showed that NPC-IFRs vs. NPC-NPDs could be differentiated by 8 features (AUCs: 0.727–0.835). The classification models showed potential in prediction of NPC-IFR with higher accuracies (ANN: 0.812, KNN: 0.775, SVM: 0.732).

**Conclusion:** In-field and high-dose region relapse were the main recurrence patterns which may be due to the radioresistance. After integration in the clinical workflow, radiomic analysis can be served as imaging biomarkers to facilitate early salvage for NPC patients who are at risk of in-field recurrence.

## Introduction

The incidence and mortality of nasopharynx cancer (NPC) were estimated to 130,000 and 73,000 worldwide, respectively, in 2018 (1). In China, 60.600 cases were diagnosed with NPC in 2015, responsible for approximately 1.41% of the total incidence of malignancy (2).

Intensity-modulated radiotherapy (IMRT) has served as a major breakthrough in the treatment of head and neck cancer carcinoma (HNC) as it is capable of providing a highly conformal dose distribution with sharp dose gradients. By conforming the doses to the irregular tumor, dose escalation is possible with IMRT, which has greatly improved the therapeutic ratio and local control after radiotherapy (3). However, local-regional recurrence remains the major cause of treatment failure in patients with NPC (4). Although some research has been carried out on the patterns of local-regional recurrence (e.g., out of field, in field, marginal miss) (4–8), few studies have focused on early identification of patients who are at higher risk of in-field recurrence before radiotherapy. Therefore, new tools are needed for further investigation of radiation resistant of NPCs with in-field recurrence.

Radiomics is a new research field to decode tumor phenotype by quantitative analysis of image features extracted from medical images, with the goal of personalized clinical decision making and improving patient stratification. This advanced technology has shown the significant predictive power for gene expression, pathological classification, response to treatment, and prognosis (9–13). By extracting a high-dimensional mineable feature set from MRI images, recent studies on NPC have found that the features are associated with pathological types, progression free survival (PFS), local or distant treatment failure, and treatment response (14). Thus far, to our knowledge, although prior studies (4–8) have been able to explore the recurrence patterns of failure, further radiomic analysis for in-field recurrence has not been performed at present.

In this study, we investigated the potential of MRI-based radiomics in characterizing radioresistance of nasopharyngeal carcinoma (NPC) with in-field recurrence. Specifically, we examined whether radiomic features could distinguish NPC-IFR (NPC with in-field recurrence) from NPC-NPD (NPC with non-progression disease).

## Materials and Methods

### Patients and Tumor Characteristics

This retrospective study was approved by the Nanjing Drum Tower Hospital's ethics committee, and informed consent was waived. Three hundred and six patients administered radiation with IMRT for NPC from July 2009 to August 2016 were reviewed. Inclusion criteria were: (a) biopsy-proven nasopharyngeal carcinoma; (b) absence of metastases; (c) developed with recurrence after IMRT; (d) radiotherapy (RT) for primary disease were administered at Nanjing Drum Tower Hospital; (e) CT, and/or MRI/PET examination were performed before and after RT; (f) completion of planned radiation treatment; (g) follow-up of more than 3 months.

A total of 20 recurrent NPCs who met the criteria were identified. Table 1 shows the general characteristics of the patients.

**Table 1:** Baseline characteristics of 20 NPCs with recurrence.

**Characteristic**	**Number of patients (%)**
Total	20
**Gender**	
Male	16/20 (80%)
Female	4/20 (20%)
**Age (years)**	
Median (range)	51 (41–66 years)
**AJCC staging**	
III	17/20 (85%)
IVa	3/20 (23%)
**Concurrent Chemotherapy**	
Yes	20/20 (100%)
No	0/20 (0%)
Radiotherapy technique	IMRT

### Imaging Method

Pretreatment and recurrent MR images were obtained with a 3.0 T MRI unit (Achieva 3.0T X-series, Philips Healthcare, Best, Netherlands) according to a standard clinical acquisition protocol: SPAIR T2W MRI (repetition time [TR], 3,000 ms; echo time [TE], 100 ms; flip angel, 90 degrees; matrix size, 212 × 141; slice thickness 4 mm, in-plane resolution 0.65 mm × 0.65 mm). SPAIR is a kind of fat-suppression techniques which is desirable to remove the fat contribution from MR imaging signal to better visualize pathology or contrast enhanced (15). Tie et al. (16) reported that the addition of fat suppression techniques to T2W MR sequences improves the detection and delineation of head and neck lesions.

### Target Delineation and IMRT Treatment Planning

Patients were treated with supine position and immobilized by a thermoplastic head and shoulder mask. CT images with slice thickness of 3 mm were obtained and transferred to Pinnacle software (Philips Medical Systems, Andover, MA, United States) for treatment planning design.

Tumor volumes were defined by both experienced radiologists and radiation oncologists on simulation CT images registered with MRI images. The gross tumor volume (GTV) including primary gross tumor volume (GTVp) and involved lymph nodes (GTVnd), was defined as the visible tumor based on imaging, clinical examination, as well as endoscopic findings. The elective clinical target volume (CTV) was defined to include regions and lymph nodes that have a high risk of tumor involvement. The planning target volume (PTV) was constructed automatically by expanding the corresponding CTV by 3–5 mm according to the immobilization and localization uncertainties.

The range of the total prescribed RT dose was 70–74 Gy. All patients were treated at 2–2.2 Gy daily fractions, one fraction per day, 5 days per week, as shown in Table 2. The radiation dose was prescribed to cover at least 96% of PTV. All plans were assessed and confirmed by both senior physicians and oncologists. All patients were treated with external-beam radiation therapy using 6-MV photons, 7–9 radiation fields.

**Table 2:** Details of recurrent patients and their failure patterns.

**No**.	**Primary tumor site**	**Stage**	**RT dose (Gy/fr)**	**Concurrent chemotherapy**	**Time to failure (Months)**	**Site of recurrence**	**Patterns of failure**
1	Nasopharynx	III	50 Gy/25 + 20 Gy/10	Nedaplatin	19	Local	In field
2	Nasopharynx	III	50 Gy/25 + 20 Gy/10	Paclitaxel liposome	65	Regional	In field
3	Nasopharynx*	III	44 Gy/20 + 22 Gy/10 + 4 Gy/2	Nedaplatin	14	Local	In field
4	Nasopharynx	III	44 Gy/20 + 22 Gy/10 + 4 Gy/2	Paclitaxel liposome	49	Regional	Marginal
5	Nasopharynx*	III	50 Gy/25 + 20 Gy/10	Nedaplatin	30	Local-regional	In field
6	Nasopharynx*	III	50 Gy/25 + 20 Gy/10	Nedaplatin	17	Local-regional	In field
7	Nasopharynx	III	44 Gy/20 + 22 Gy/10 + 4.4 Gy/2	Nedaplatin	54	Regional	Out of field
8	Nasopharynx*	III	50 Gy/25 + 20 Gy/10	Nedaplatin	8	Local	In field
9	Nasopharynx*	III	44Gy/20 + 22 Gy/10 + 4.4 Gy/2	Docetaxel	34	Local-regional	In field
10	Nasopharynx*	IVa	44 Gy/20 + 22 Gy/10 + 6.6 Gy/3	Nedaplatin	26	Local	In field
11	Nasopharynx	III	66 Gy/30 + 4 Gy/2	Docetaxel+oxaliplatin	27	Local	In field
12	Nasopharynx*	III	50 Gy/25 + 20 Gy/10	Nedaplatin	11	Regional	In field
13	Nasopharynx*	III	50 Gy/25 + 20 Gy/10	Docetaxel	41	Regional	In field
14	Nasopharynx	IVa	50 Gy/25 + 20 Gy/10 + 4 Gy/2	Cetuximab+Nedaplatin	26	Local	In field
15	Nasopharynx*	III	50 Gy/25 + 20 Gy/10	Nedaplatin	26	Regional	In field
16	Nasopharynx	IVa	44 Gy/20 + 22 Gy/10 + 4.4 Gy/2	Docetaxel	51	Regional	In field
17	Nasopharynx	III	50 Gy/25 + 20 Gy/10	Oxaliplatin	41	Regional	In field
18	Nasopharynx*	III	44 Gy/20 + 22 Gy/10 + 4.4 Gy/2	Nedaplatin	33	Local	In field
19	Nasopharynx*	III	50 Gy/25 + 20 Gy/10	Paclitaxel liposome	14	Local	In field
20	Nasopharynx	III	50 Gy/25 + 20 Gy/10 + 4 Gy/2	Nedaplatin	23	Local	In field

* 11 NPC-IFRs (in field recurrence) with pre-treatment MRI images available are subsequently used for radiomic analysis

### Chemotherapy

The chemotherapy program was performed according to clinicians' assessment of multi-factors, including age, comorbidity, contraindication, tumor extent and social support. One patient (No.10 in Table 2) in stage IVa received neoadjuvant chemotherapy with nedaplatin + 5-fluorouracil. All patients received nedaplatin/docetaxel/paclitaxel liposome based concurrent chemotherapy. Adjuvant chemotherapy was delivered to all patients following concurrent chemoradiation. The most common regimen of adjuvant chemotherapy included nedaplatin + 5-fluorouracil or docetaxel + 5-fluorouracil or nedaplatin + 5-fluorouracil+docetaxel or paclitaxel + gemcitabine (17–20).

### Image Registration and Recurrence Definition

Local recurrence referred to the disappearance of the primary tumor treated with radical radiation but the presence of new lesions later, while regional recurrence referred to the reappearance of metastatic lymph nodes in the lymphatic drainage area. The local regional recurrences were confirmed by MRI and/or PET scan, or pathological biopsy examination if applicable.

For patients with local regional failure, MRI and/or PET scans obtained at the time of recurrence were registered with pretreatment planning CT, by rigid registration first and then deformable registration and visual assessment, using MIM (MIM Software, Inc., Cleveland, OH, United States). The recurrent tumor volume (V_recur_) was subsequently identified and delineated by the user blinded to the original tumor volume and isodose distribution.

After V_recur_ was delineated on the pretreatment planning CT, the radiation dosed received by V_recur_ was then obtained by analyzing the dose-volume histogram (DVH). Depending on the position of V_recur_, the recurrences were classified into occurring inside or outside the high-dose target volume: “in field,” in which 95% or more of V_recur_ was within the 95% prescription isodose; “marginal miss,” if 20 to 95% of the V_recur_ was inside the 95% prescription isodose; “out of field,” if < 20% of the V_recur_ was within the 95% prescription isodose (8).

**Figure 1:**
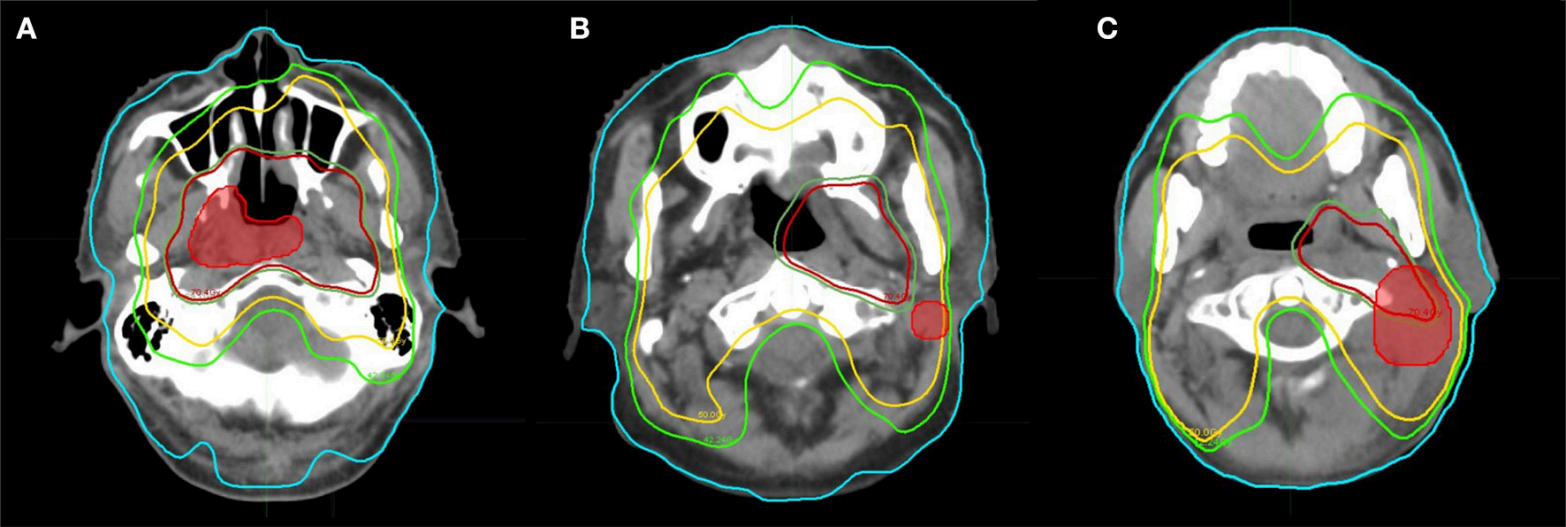
Patterns of failure for patients with recurrence, with the accumulated dose and site of recurrence. **(A)** In field. **(B)** Out of field. **(C)** Marginal.

Recurrences were defined as local if the failures occurred inside the primary tumor, and as regional if the failures occurred elsewhere including neck lymph nodes. Figure 1 shows the type of failures for patients with recurrences.

### Image Preprocessing and Radiomic Feature Extraction for NPC

**Figure 2:**
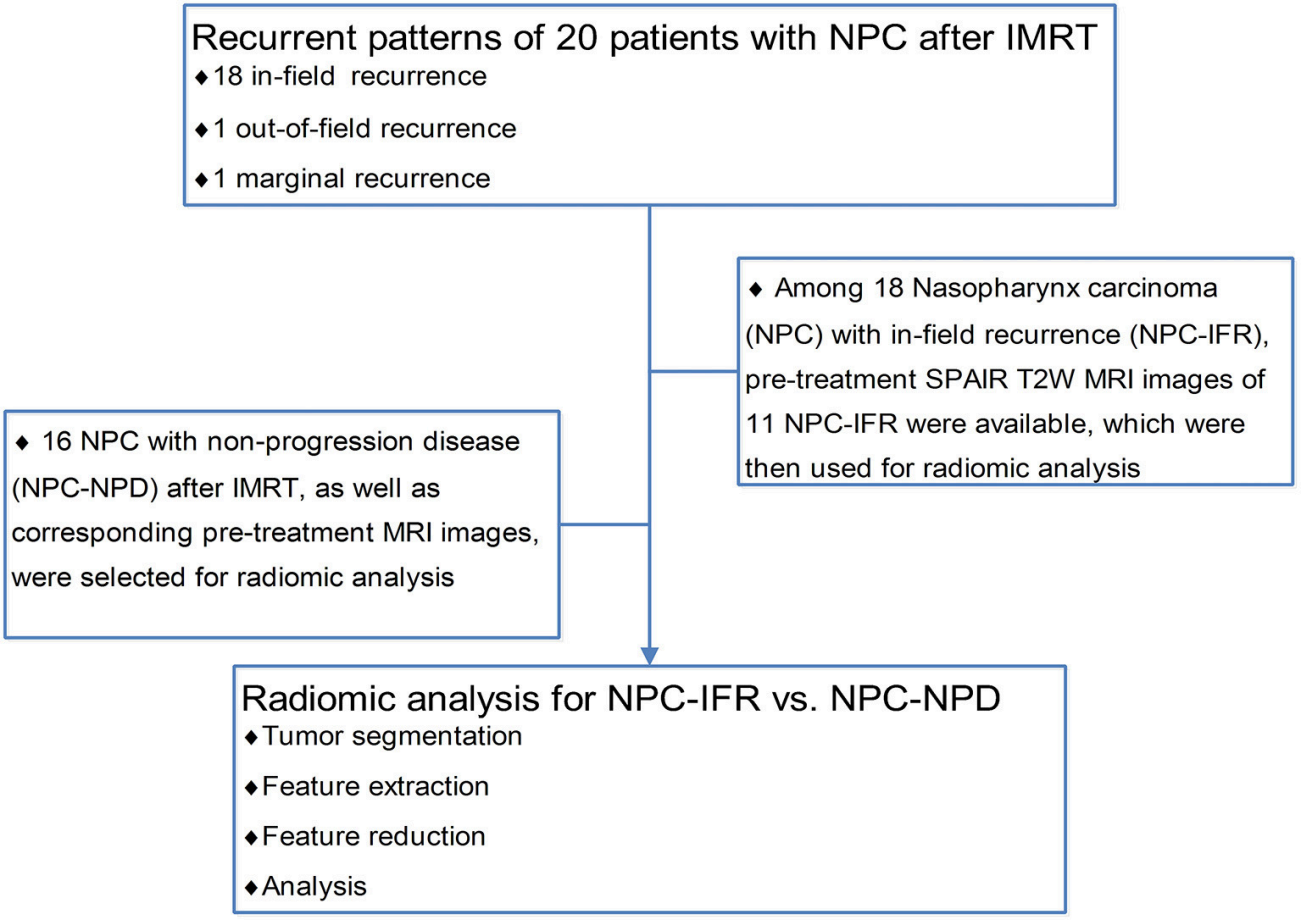
Flowchart of using radiomic analysis in recurrent pattern.

To investigate the feasibility of radiomics for analysis of NPC radioresistance, NPCs with in-field recurrences (NPC-IFR) and with non-progression disease (NPC-NPD) after treatment were enrolled for radiomic analysis. NPC-NPD were met the following inclusion criteria: (a) biopsy-proven nasopharyngeal carcinoma; (b) absence of metastases; (c) RT for primary disease were administered at our institution; (d) completion of planned radiation treatment; (e) followed-up for more than 36 month and has not been lost to date; (f) available of pretreatment SPAIR T2W MR images. A total of 16 recurrent NPC-IFR who met the criteria were identified. Figure 2 shows the flowchart of using radiomic analysis in NPC-IFR vs. NPC-NPD.

The radiomic features were extracted from pretreatment gross tumor volume. Before using these treatment-planning contours for radiomic research, each contour was manually modified to avoid adjacent air, fat and surrounding organs by two senior board-certified radiation oncologists. In addition, for each contour, gray-level normalization was performed, using a method that normalize the image intensities in a range of [*m* – 3*s, m* + 3*s*] (*m*, mean value of gray-level in the contours; *s*, standard deviation of gray-level), to minimize the influence of contrast and brightness variation (21). The gray levels that were located outside the range [*m* ± 3*s*] were excluded for further analysis and the range obtained was subsequently quantized to 6 bits (between 1 and 64) (21). Moreover, the contoured volume with voxel size of 0.65 × 0.65 × 4 mm^3^ were resampled to a voxel size of 1 × 1 × 1 mm^3^ using cubic interpolation algorithm before feature extraction to unify the voxel size across the cohort.

Radiomic feature computation was performed using pyradiomics V1.3.0 (22). Pyradiomics is an open-source python package for the extraction of radiomic features from two-dimensional (2D) or three-dimensional (3D) medical imaging data. With this package, the following methods were used for feature computation: (1) morphological features: descriptors of shape and size; (2) intensity histogram (IH): describe the distribution of voxel intensities within the contoured volume; (3) five texture matrices: *a*. gray-level co-occurrence matrix (GLCM, 13 angles in 3D [26-connectivity], distance = 1 voxel); *b*. gray level size zone matrix (GLSZM, 13 angles in 3D, distance = 1 voxel); *c*. gray level run length matrix (GLRLM, 13 angles in 3D); *d*. neighboring gray tone difference matrix (NGTDM, neighborhood size: 3 × 3 × 3 voxels); *e*. gray level dependence matrix (GLDM, distance = 1 voxel). As for GLCM, GLSZM, and GLRLM, each feature was calculated on each angle separately, after which the mean value of the feature was obtained. (4) Laplacian of Gaussian (LoG)-filtration and Wavelet-transform features: all the aforementioned texture matrices can also be calculated on a derived image, obtained by applying LoG band pass filter and wavelet filter on the original image. In particular, LoG filter was applied on the contoured volumes for enhancing fine to coarse texture (filter width: fine, σ = 0.5; medium, σ = 1.5; coarse, σ = 2.5) and wavelet filter was applied to focus features on the different decomposition and approximation level of the original contoured volumes.

**Figure 3:**
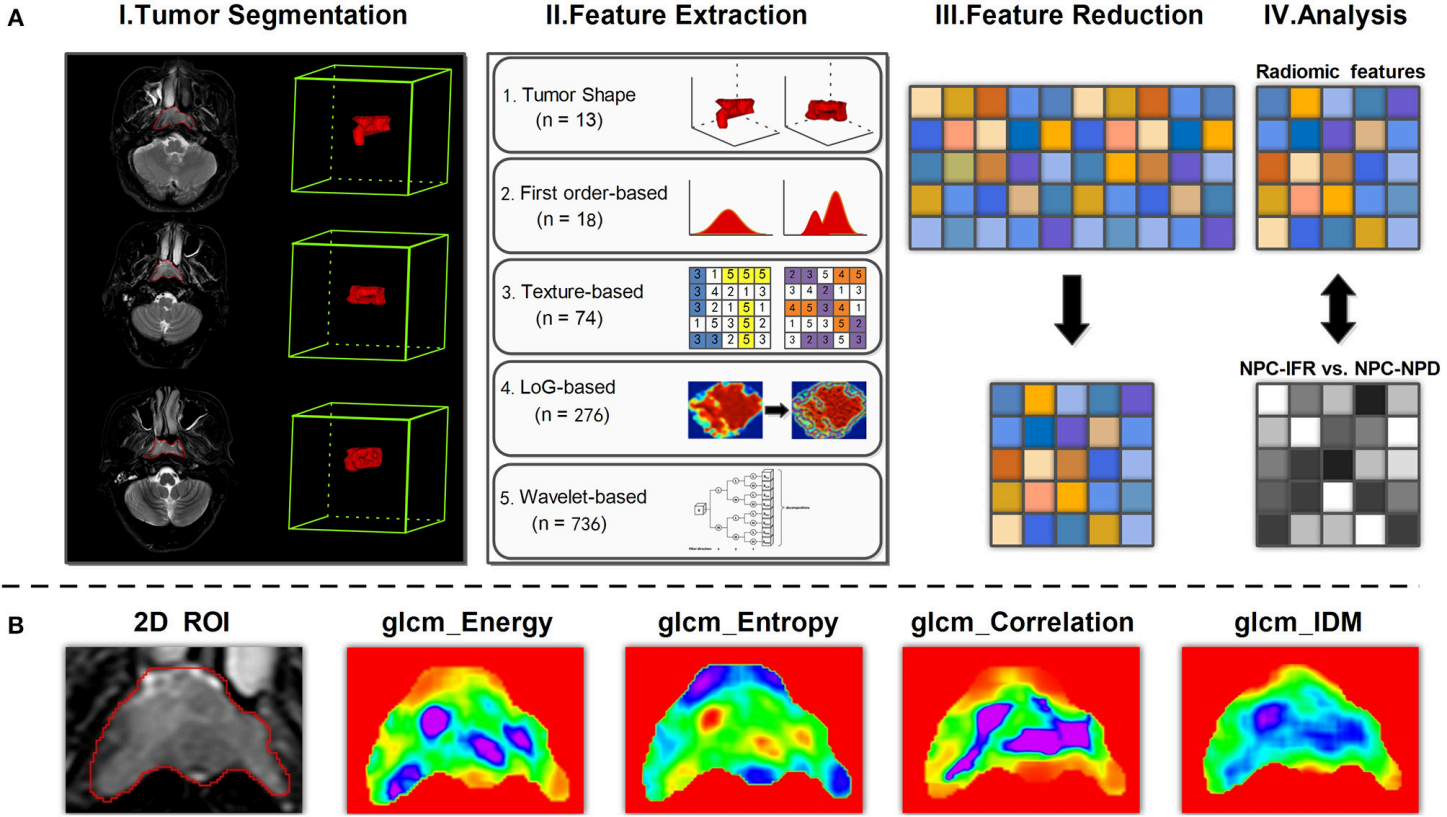
**(A)** Workflow of radiomic analysis for discrimination between NPC-IFR (NPC with in-field recurrence) and NPC-NPD (NPC with non-progression disease). I, Image segmentation was performed on SPAIR T2W MR images. II, Features were extracted from the tumor contours on the MR images using shape, first order, texture, LoG and wavelet-based method. III, Principal component analysis (PCA) was performed on significant features for dimension reduction. IV, For the analysis, principal components derived from significant features were combined with supervised machine learning method for prediction of NPC-IFR vs. NPC-NPD. **(B)** Examples of feature maps computed from two-dimensional tumor region by using GLCM method (e.g., Energy, Entropy, Correlation, InverseDifferenceMoment [IDM]).

Overall, as for each contoured volume, quantitative radiomic features were calculated from original image and derived image, using five principal algorithms: morphological-based (shape and size), IH-based (intensity histogram), texture-based (GLCM, GLSZM, GLRLM, GLDM), LoG filter-based (LoG_σ = 0.5/1.5/2.5__GLCM, LoG_σ = 0.5/1.5/2.5__GLSZM, LoG_σ = 0.5/1.5/_2.5_GLRLM, LoG_σ = 0.5/1.5/2.5__GLDM), andWavelet transform−based (Wavelet_level__GLCM, Wavelet_level__GLSZM, Wavelet_level__GLRLM, Wavelet_level__GLDM). A complete list of the features was shown in Supplementary Table S1. Figure 3A shows the workflow of radiomic analysis for NPC-IFR. Figure 3B shows the example of GLCM textural feature maps calculated from 2D slices.

### Statistical Analysis

Statistical analysis was performed using R statistical and computing software version 3.3.2 (http://www.rproject.org, R Foundation for Statistical Computing, Vienna, Austria).

Inter-observer variability in measurement of MRI radiomic features was evaluated by calculating intraclass correlation coefficients (ICC) (ICC < 0.40, poor; 0.40 ≤ ICC < 0.60, moderate; 0.60 ≤ ICC < 0.80, good; ICC ≥ 0.80, excellent), using “irr” package (ver. 0.84 in R software). It was calculated for assessment of feature reproducibility in repeated delineation and then features with ICC < 0.8 were removed. Additionally, for the aforementioned selected features (ICC ≥ 0.80), we also compute Pearson correlation coefficient (PCC) by conducting a correlation matrix, to quantify the pair-wise correlations. In this work, for example, if two features appeared a strong correlation (|PCC| ≥ 0.80), we look at the mean absolute correlation coefficient of each feature (with the remaining features) and remove the feature with the largest mean absolute correlation. Using the above methods, influential feature sets were generated with high reproducibility and low redundancy.

Statistical significance of each influential feature for discrimination between NPC-IFR and NPC-NPD was analyzed using Kruskal-Wallis test. The Benjamini-Hochberg method was performed to correct *P*-values for multiple testing. *P* < 0.05 were considered significant. Diagnostic performance of the significant features was assessed by using receiver operating characteristic (ROC) curve and the area under the curve (AUC) analysis. All the significant features were selected for further analysis.

### Feature Reduction and Radiomic Machine-Learning Classifiers

Prior to classification, principal component analysis (PCA) was used to further reduce the feature vector dimensions and to increase the discriminative capability. Those principal components that sufficiently accounted for 85% of the significant feature subset variability were selected for further modeling. Next, supervised machine-learning classifiers (ANN, KNN, and SVM) were then established and validated with the stratified 10-fold cross-validation in Weka (University of Waikato, Hamilton, New Zealand) to evaluate how well these predictive models would perform with the subset of the components derived from PCA method. The associated metrics including false positives (FP), true positives (TP), accuracy, and matthews correlation coefficient (MCC) were calculated for model evaluation.

## Results

### Patterns of Recurrence

A total of 20 NPC patients with recurrence were met the inclusion criteria. During a median follow up period of 26.5 months, 9 patients had local recurrences, 8 patients had regional recurrences, and 3 patients had local-regional recurrences. As for the patterns of recurrence, 18 (90%), 1 (5%), and 1 (5%) patients were identified as in-field failures, marginal, and out-of-field failures, respectively. Details of recurrent patients and their local or/and regional failure are summarized in Table 2. As shown in Table 2, 18 NPCs developed in-field recurrences (NPC-IFR). Among these 18 NPC-IFRs, 11 cases with pre-treatment MRI images available were then used for radioresistance analysis by radiomics method.

### Predictive Capabilities of Radiomic Features for NPC-IFR

**Figure 4:**
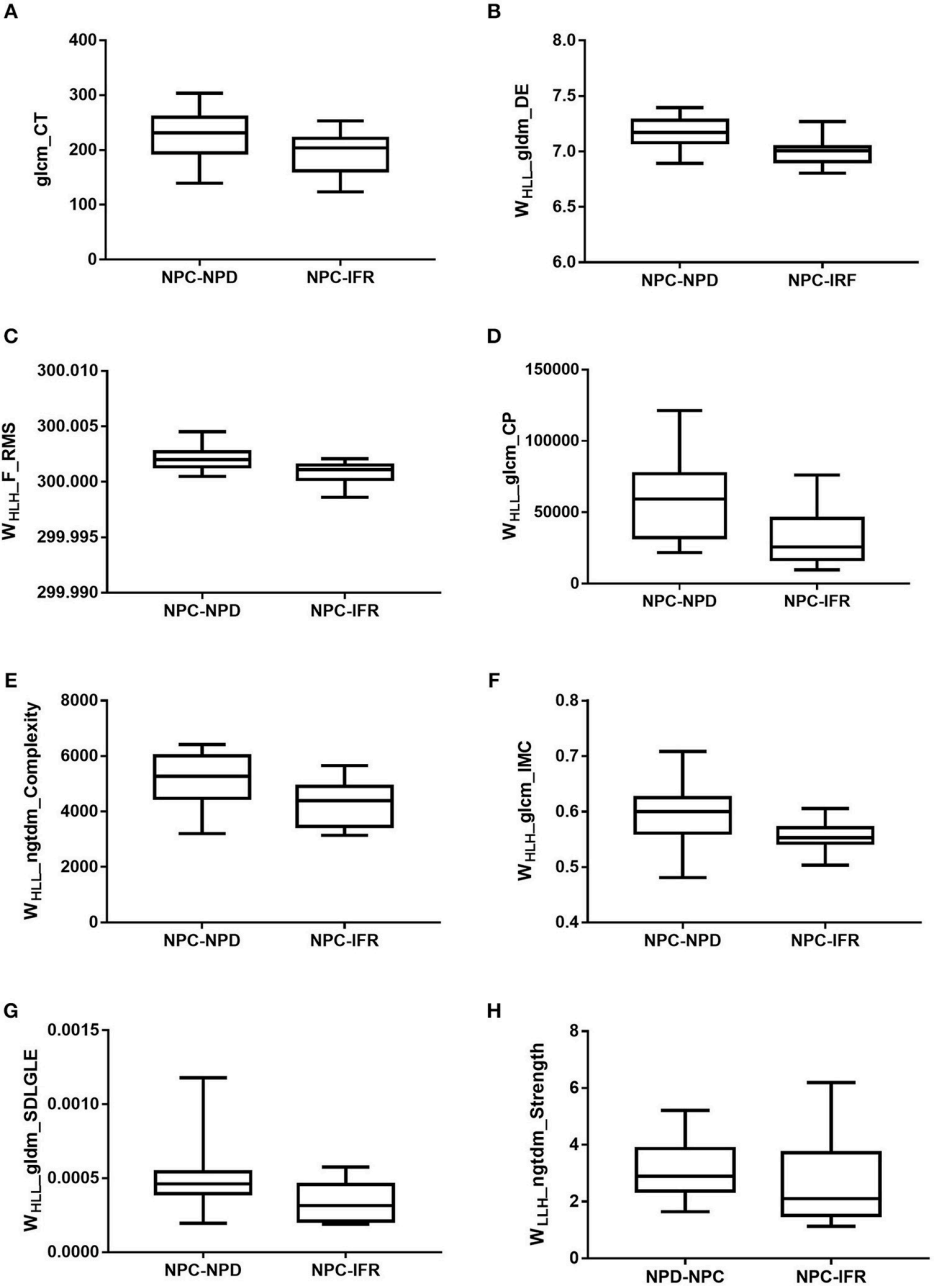
Box plots of amplitude features, successfully differentiating NPC-IFR from NPC-NPD. **(A)** glcm_CT (*P* = 0.046); **(B)** WHLL_gldm_DE (*P* = 0.023); **(C)** WHLH_F_RMS (*P* = 0.023); **(D)** WHLL_glcm_CP (*P* = 0.032); **(E)** WHLL_ngtdm_Complexity (*P* = 0.041); **(F)** WHLH_glcm_IMC (*P* = 0.041); **(G)** WHLL_gldm_SDLGLE (*P* = 0.048); **(H)** WLLH_ngtdm_Strength (*P* = 0.048).

**Figure 5:**
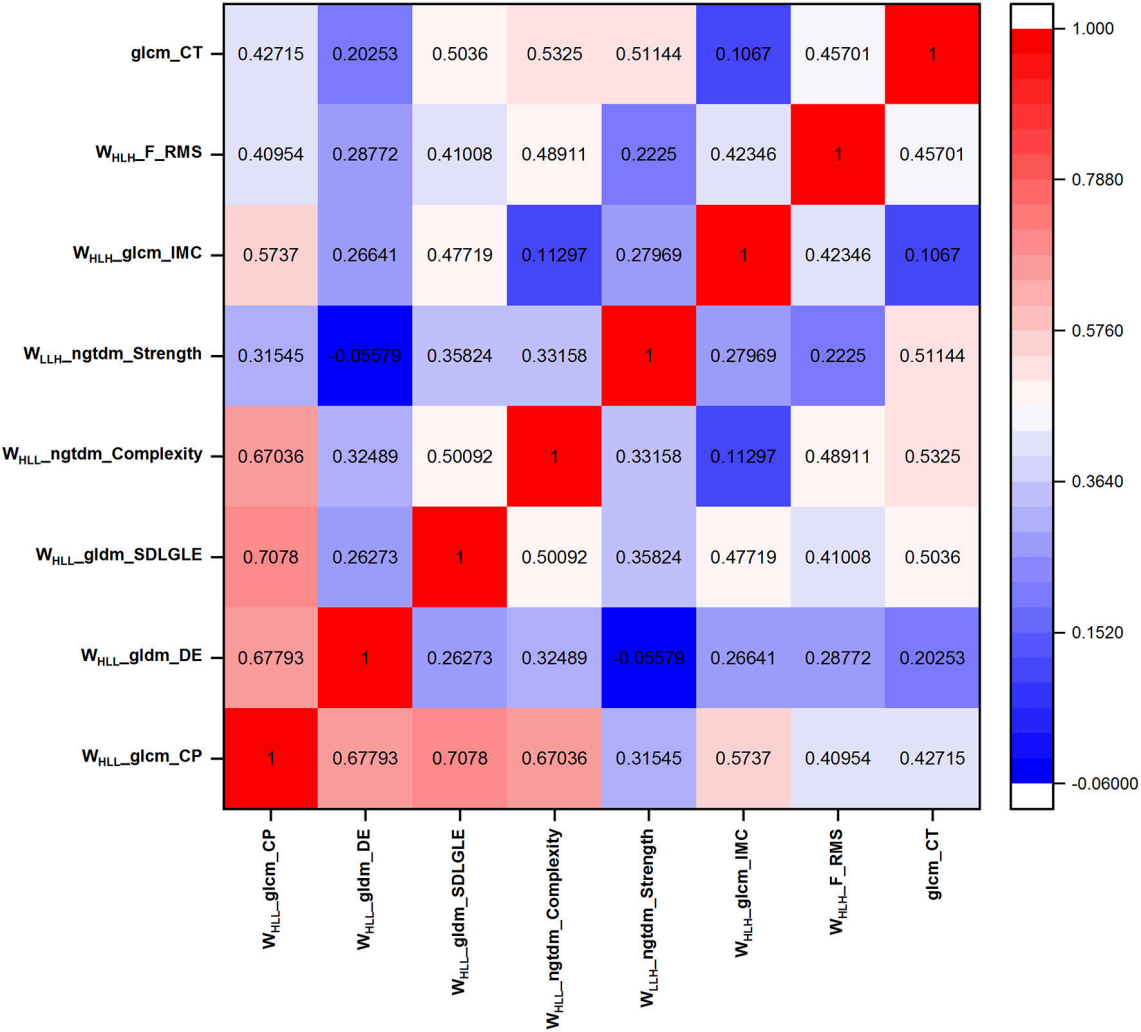
Pearson correlation coefficient of the eight significant features.

**Figure 6:**
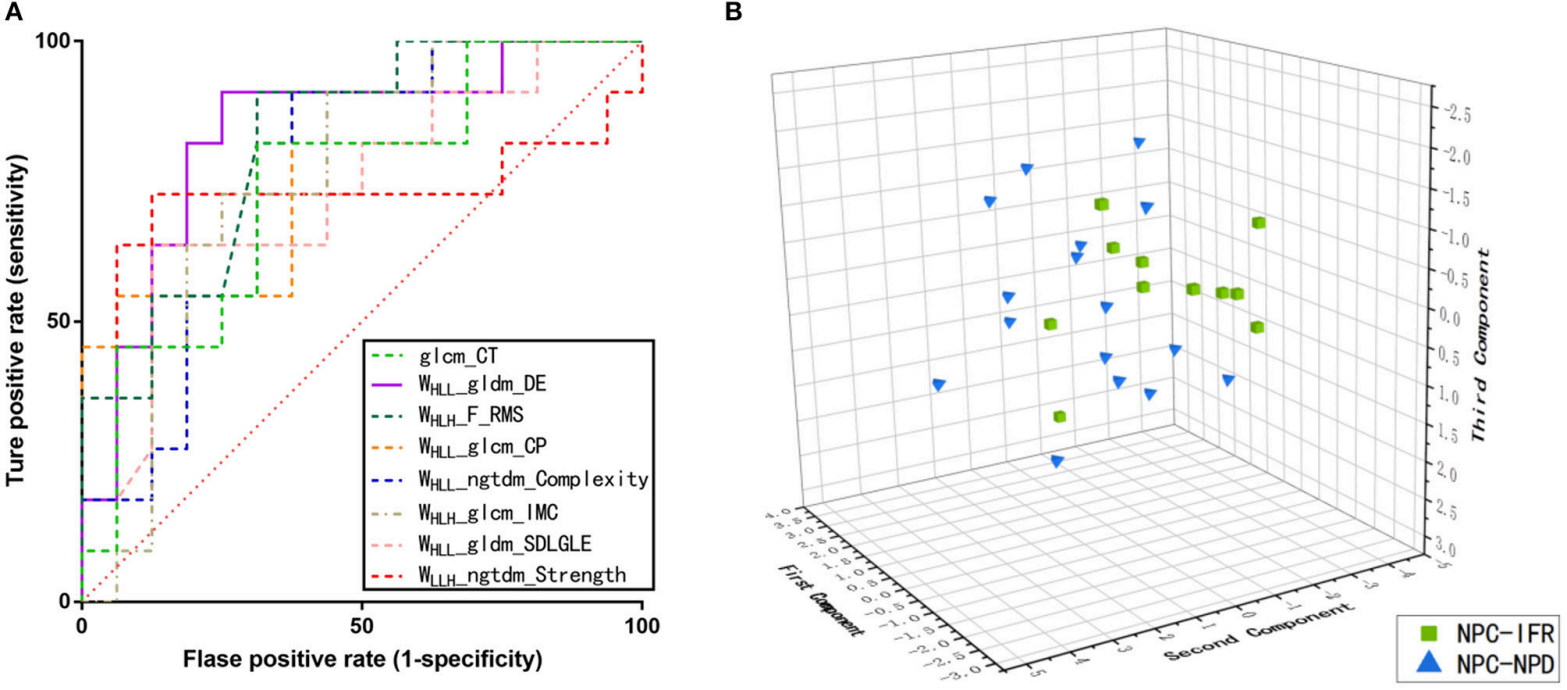
**(A)** Receiver operating characteristics (ROC) curves on the basis of the significant features. **(B)** Three-dimensional scatter plot of the NPC-IFR and NPC-NPD by using three principal components derived from the above eight significant features.

In addition to these 11 patients with NPC-IFR, 16 NPC patients with non-progression disease (NPC-NPD), as well as the corresponding MR images were also enrolled in this study for exploring the predictive power of the imaging features. Supplementary Table S2 showed the general characteristics of NPC-NPDs. A total of 1117 imaging features, preprocessed with or without LoG_σ = 0.5/1.5/2.5_ and wavelet filter, were computed from each of the 27 cases (11 NPC-IFR, 16 NPC-NPD). Of the complete radiomic feature set, influential features were yielded by calculating ICC and PCC values. Results of the Kruskal-Wallis test revealed that 8 features (1 texture feature, 7 wavelet feature; *P*-value: 0.023–0.048) were capable of differentiating between NPC-IFR and NPC-NPD. Table 3 summarizes the details of the corresponding significant features and Figure 4 presents their distribution, as well as Figure 5 shows the PCC values among them. In order to assess the diagnostic performance of the significant features, ROC analysis was used and the associated AUC values were obtained (range from 0.727 to 0.835). Figure 6A displays the ROC curves of all significant features.

**Table 3:** Features show statistical difference between NPC-IFR and NPC-NPD.

**Feature**	***P* value**	**Standard error**	**95% CI**	**AUC**	**Sens**	**Spec**
glcm_CT	0.046	0.099	0.541–0.891	0.744	0.818	0.687
W_HLL__gldm_DE	0.023	0.084	0.643–0.949	0.835	0.909	0.750
W_HLH__F_RMS	0.023	0.079	0.636–0.946	0.830	0.909	0.687
W_HLL__glcm_CP	0.032	0.093	0.591–0.922	0.790	0.909	0.625
W_HLL__ngtdm_Complexity	0.041	0.094	0.559–0.903	0.761	0.909	0.625
W_HLH__glcm_IMC	0.041	0.096	0.553–0.899	0.756	0.727	0.750
W_HLL__gldm_SDLGLE	0.048	0.104	0.523–0.879	0.727	0.636	0.875
W_LLH__ngtdm_Strength	0.048	0.126	0.523–0.879	0.727	0.727	0.875

*glcm, gray-level co-occurrence matrix; CT, cluster tendency; W_HLL_, volume with a wavelet high-pass filter along x-direction, a low-pass filter along y-direction and a low-pass filter along z-direction; gldm, gray-level dependence matrix; DE, dependence entropy; RMS, root mean squared; CP, cluster prominence; ngtdm, neighboring gray-tone difference matrix; IMC, informational measure of correlation; SDLGLE, small dependence low gray Level emphasis; CI, confidence interval; AUC, area under the curve; Sens, Sensitivity; Spec, Specificity; NPC-IFR, NPC with in-field recurrences; NPC-NPD, NPC with non-progression disease*.

### Supervised Classification

After radiomic quantification and feature reduction process, we found that the first three principal components were most significant on the basis of PCA method (with the accumulated variance of the components was 85.43%). Figure 6B shows the distribution of the patients using the three components. Supervised machine-learning algorithms (ANN, KNN, and SVM) were performed on these three components and the efficiency of classification was validated by stratified 10–fold CV, with the results showed that ANN, KNN and SVM obtained the accuracies of 0.812, 0.775, and 0.732, respectively. The predictive results of the models are presented in Table 4.

**Table 4:** Summary of Classification Results Obtained from stratified 10-Fold cross-validation on two classification groups by ANN, KNN, and SVM model.

**Algorithm**	**TP rate**	**FP rate**	**Precision**	***F*-measure**	**MCC**	**Accuracy**
ANN	0.815	0.231	0.814	0.813	0.613	0.812
KNN	0.778	0.210	0.790	0.780	0.559	0.775
SVM	0.741	0.292	0.738	0.738	0.457	0.732

ANN, artificial neural network; KNN, k-nearest neighbor; SVM, support vector machine; FP, false-positive; TP, true-positive; MCC, matthews correlation coefficient

## Discussion

Understanding the failure patterns for NPC patients treated with IMRT plays a central role in radiotherapy planning optimization and patient clinical management. Prior works have documented the different failure patterns for HNC patients. Oksuz et al. (8) and Johansen et al. (7), for example, analyzed the recurrence patterns among HNC patients treated with radical (chemo-) radiotherapy. They reported that “in field” failures were the majority of recurrence. Similarly, “in field” failures are the main patterns of local-regional recurrence for NPC (4–6), suggesting that it may be related to the radioresistance of tumor cells. A review such as that conducted by Hong et al. has shown that radioresistance may be responsible for NPC local-regional recurrence (23). Therefore, new tools should be developed to quantify heterogeneity within tumors for further analysis of radioresistance. Radiomic analysis is emerging as a new method to quantify the tumor heterogeneity, Ganeshan et al. has found that texture features are associated with tumor hypoxia and angiogenesis (24). To data, there have been few studies to explore the power of radiomics for predicting “in field” failures of NPC. Promising results have been presented for predicting local failure of NPC patients using radiomic-based method (25). However, a major problem with the above research was that the therapeutic regimen and local failure pattern of NPC patients were not specified in detail. It has yet to investigate whether causes of in-field recurrence was due to insufficient tumor volume delineation or tumor heterogeneity.

This study set out with the aim of analyzing the recurrence patterns and reasons in patients with NPC treated with IMRT and investigating the predictive power of MRI-based radiomic features for radioresistance. In present study, 1 patient developed with “marginal” recurrence and 1 patient occurred with “out of field” recurrence, which may due to the risk of marginal miss for IMRT and/or inadequate target volume delineation. Moreover, “in field” failure was the major recurrence pattern (90%, 18/20) and similar to the reports from other centers (4–8). It may be questioned whether heterogeneity within tumors may cause of “in field” recurrence. Therefore, in current work, we further analyzed 1117 radiomic features extracted from tumor volumes for 27 NPC patients (11 NPC-IFRs, 16 NPC-NPDs) and found that 8 parameters were able to discriminate between NPC-IFR and NPC-NPD, with AUC values range from 0.727 to 0.835. These findings suggested that there is a significant difference within the tumor tissue between NPC patients who resisted to radiation vs. those who do not, and these potential differences can be detected and quantified by using the radiomic parameters extracted from pretreatment MRI images. The radiomic features were mathematical measurement concerned with the distribution of gray levels within the tumor region that reflects underlying pathophysiologic and phenotypic characteristics (26). In other words, the studied radiomic features in this work captured tumor heterogeneity at local macro- and micro-scale, using the data-characterization algorithms, indirectly characterizing areas of hemorrhage, necrosis, high cell density, myxoid change, and hypoxia. Thus, with this context, tumor heterogeneity induced radioresistance could be predicted by radiomic analysis. Our work presents one of the first attempts to employ the pretreatment imaging biomarkers for prediction of “in field” failures for NPC patient (NPC-IFR).

In addition, we showed as well that the radiomic features combined with machine learning algorithms was strongly predictive and validated among NPC patients (NPC-IFD vs. NPC-NPD), and was associated with radioresistance. To minimize the over-fitting or bias, we performed a series of pre-processing: feature reproducibility and correlation evaluation, as well as principal component analysis. After the pre-processing procedures and machine-learning process, the three models achieved high accuracies (ANN: 0.812; KNN: 0.775; SVM: 0.732) on the basis of the selected attributes. A possible physiologic explanation for these observations is the difference in intratumoral heterogeneity between NPC-IFD and NPC-NPD, which can be captured by radiomic features. These findings extend those of Zhang et al. (25), we not only analyzed the recurrence patterns for NPC patients but also demonstrated that machine-learning models trained with the radiomic features described have the potential to predict the risk of NPC patients developing “in field” recurrence. This work thus indicates the benefit gained form radiomic analysis could potentially speed up the development of personalized medicine.

Several limitations are worth noting in this work, namely the small patient cohort in a single center and the retrospective nature of the analysis. Due to the relative small simple size, an independent external validation of the radiomic models was not performed. Future prospective study is needed to verify the work with a much larger prospective cohort of patients. In addition, although recent radiomic works have shown the promising results in different cancers, the radiomic-biology correlations have not yet to be determined. Therefore, other extensions of this work would be to incorporate the biological data of tumor, to explore the potential mechanism further.

In conclusion, MR imaging-based radiomic features combined with supervised machine-learning algorithms were found to early discriminate NPC patients with or without “in field” recurrence before IMRT. These findings suggest the potential value for radiomics to provide a quantitative, objective measurement of NPC patients who had a higher risk to develop “in field” recurrence, with advantage of low cost, using existing MR imaging data, without subjecting patients to extra radiation exposure or imaging.

## Ethics Statement

Ethical approval was given by the medical ethics committee of Nanjing Drum Tower Hospital with the following reference number No. 2018-016-09.

## Author Contributions

SL, KW, and ZH: Conception and design. JuY and JL: Administrative support. JiY, BL, WR, SG, JL, and FM: Provision of study materials or patients. SL, KW, ZH, and WR: Collection and assembly of data. SL and ZH: Data analysis and interpretation. All authors manuscript writing and final approval of manuscript.

### Conflict of Interest Statement

The authors declare that the research was conducted in the absence of any commercial or financial relationships that could be construed as a potential conflict of interest.
