# Supplementary material for: Use of Radiomics Combined With Machine Learning Method in the Recurrence Patterns After Intensity-Modulated Radiotherapy for Nasopharyngeal Carcinoma: A Preliminary Study
Source: Front Oncol. 2018 Dec 21;8:648. doi: 10.3389/fonc.2018.00648 (PMC6308146; doi:10.3389/fonc.2018.00648)
Supplement: Supplementary file 1 [file Data_Sheet_1.docx]

Table S1. Feature Type and Associated Features

| Feature Type | Methods | Feature name |
| --- | --- | --- |
| Morphology -based |  | Maximum3Ddiameter (M3D)  Maximum2DDiameterSlice (M2DS)  Sphericity  MinorAxis(MA)  Elongation  SurfaceVolumeRatio (SVR)  Volume  MajorAxis(MA1)  SurfaceArea(SA)  Flatness  LeastAxis(LA)  Maximum2DDiameterColumn (M2DC)  Maximum2DDiameterRow (M2DR) |
| First order-based | Histogram | InterquartileRange (IQR)  Skewness  Uniformity  Median  Energy  RobustMeanAbsoluteDeviation (RMAD)  MeanAbsoluteDeviation (MAD)  TotalEnergy(TE)  Maximum  RootMeanSquared (RMS)  90Percentile  Minimum  Entropy  Range  Variance  10Percentile  Kurtosis  Mean |
| Texture-based | GLCM | JointAverage(JA)  SumAverage(SA)  JointEntropy(JE)  ClusterShade(CS)  MaximumProbability(MP)  Idmn  JointEnergy(JE)  Contrast  DifferenceEntropy(DE)  InverseVariance(IV)  DifferenceVariance(DV)  Idn  Idm  Correlation  Autocorrelation  SumEntropy(SE)  SumSquares(SS)  ClusterProminence(CP)  Imc2  Imc1  DifferenceAverage(DA)  Id  ClusterTendency(CT) |
|  | GLSZM | GrayLevelVariance(GLV)  ZoneVariance(ZV)  GrayLevelNonUniformityNormalized(GLNUN)　SizeZoneNonUniformityNormalized(SZNUN)  SizeZoneNonUniformity(SZNU)  GrayLevelNonUniformity(GLNU)  LargeAreaEmphasis(LAE)  SmallAreaHighGrayLevelEmphasis(SAHGLE)  ZonePercentage(ZP)  LargeAreaLowGrayLevelEmphasis(LALGLE)  LargeAreaHighGrayLevelEmphasis(LAHGLE)  HighGrayLevelZoneEmphasis(HGLZE)  SmallAreaEmphasis(SAE)  LowGrayLevelZoneEmphasis(LGLZE)  ZoneEntropy(ZE)  SmallAreaLowGrayLevelEmphasis(SALGLE) |
|  | GLRLM | ShortRunLowGrayLevelEmphasis(SRLGLE)  GrayLevelVariance(GLV)  LowGrayLevelRunEmphasis(LGLRE)  GrayLevelNonUniformityNormalized(GLNUN)  RunVariance(RV)  GrayLevelNonUniformity(GLNU)  LongRunEmphasis(LRE)  ShortRunHighGrayLevelEmphasis(SRHGLE)  RunLengthNonUniformity(RLNU)  ShortRunEmphasis(SRE)  LongRunHighGrayLevelEmphasis(LRHGLE)  RunPercentage(RP)  LongRunLowGrayLevelEmphasis(LRLGLE)  RunEntropy(RE)  HighGrayLevelRunEmphasis(HGLRE)  RunLengthNonUniformityNormalized(RLNUN) |
|  | NGTDM | Coarseness  Complexity  Strength  Contrast  Busyness |
|  | GLDM | GrayLevelVariance(GLV)  HighGrayLevelEmphasis(HGLE)  DependenceEntropy(DE)  DependenceNonUniformity(DNU)  GrayLevelNonUniformity(GLNU)  SmallDependenceEmphasis(SDE)  SmallDependenceHighGrayLevelEmphasis(SDHGLE)  DependenceNonUniformityNormalized(DNUN)  LargeDependenceEmphasis(LDE)  LargeDependenceLowGrayLevelEmphasis(LDLGLE)  DependenceVariance(DV)  LargeDependenceHighGrayLevelEmphasis(LDHGLE)  SmallDependenceLowGrayLevelEmphasis(SDLGLE)  LowGrayLevelEmphasis(LGLE) |
| LoG-based | First-order statistic and texture of Laplacian of Gaussian (LoG).  Filter width: fine, σ=0.5; medium, σ=1.5; coarse, σ=2.5 | First-order features  GLCM features  GLSZM features  GLRLM features  NGTDM features  GLDM features |
| Wavelet-based | First-order statistic and texture of wavelet decomposition.  Decomposition levels: LLL, LLH, LHL, LHH, HLL, HLH, HHL, HHH. | First-order features  GLCM features  GLSZM features  GLRLM features  NGTDM features  GLDM features |

GLCM, [gray-level co-occurrence matrix], describe the second-order joint probability function of the voxel intensities within the contoured volume; GLSZM, [gray-level size-zone matrix], quantify the number of connected voxels within the contoured volume that share the same gray level intensity; GLRLM, [gray-level run-Length matrix], quantify the number of consecutive voxels that have the same gray level value; NGTDM, [neighboring gray-tone difference matrix], quantify the difference between a gray value and the average gray value of its neighbors within 3×3×3 voxels neighborhood window; GLDM, [gray-level dependence matrix], quantify the gray level dependencies in the contoured volume which is defined as the number of connected voxels within a specific distance that are dependent on the center voxel; Decomposition levels , i.e. LLH interpreted as the high-pass sub band, resulting from directional filtering of the volumn with a low-pass filter along x-direction, a low pas filter along y-direction and a high-pass filter along z-direction

Table S2. Baseline characteristics of NPC patient with non-progression disease (NPC-NPD) after IMRT.

| Characteristic | Number of patients (%) |
| --- | --- |
| Total  Gender  Male  Female | 16  11/16 (69%)  5/16 (31%) |
| Age (years)  Median (range)  AJCC staging  III  IVa | 48 (39-70 years)  15/16 (94%)  1/16 (6%) |
| Concurrent Chemotherapy |  |
| Yes  No | 16/16 (100%)  0/16 (0%) |
| Radiotherapy technique | IMRT |
| Radiotherapy dose  Median | 70Gy (70Gy-74Gy) |
